# Comparison of Crop Trait Retrieval Strategies Using UAV-Based VNIR Hyperspectral Imaging

**DOI:** 10.3390/rs13091748

**Published:** 2021-04-30

**Authors:** Asmaa Abdelbaki, Martin Schlerf, Rebecca Retzlaff, Miriam Machwitz, Jochem Verrelst, Thomas Udelhoven

**Affiliations:** 1 Environmental Remote Sensing and Geoinformatics Department, Trier University, 54286 Trier, Germany; 2 Soils and Water Science Department, Faculty of Agriculture, Fayoum University, Fayoum 63514, Egypt; 3 Environmental Sensing and Modelling, Environmental Research and Innovation Department, Luxembourg Institute of Science and Technology (LIST), L-4422 Belvaux, Luxembourg; 4 Image Processing Laboratory (IPL), University of Valencia, Parc Cientific, 46980 Paterna, Spain

**Keywords:** LUT-based inversion, hybrid method, statistical method, leaf area index, fractional vegetation cover, canopy chlorophyll content

## Abstract

Hyperspectral cameras onboard unmanned aerial vehicles (UAVs) have recently emerged for monitoring crop traits at the sub-field scale. Different physical, statistical, and hybrid methods for crop trait retrieval have been developed. However, spectra collected from UAVs can be confounded by various issues, including illumination variation throughout the crop growing season, the effect of which on the retrieval performance is not well understood at present. In this study, four retrieval methods are compared, in terms of retrieving the leaf area index (LAI), fractional vegetation cover (fCover), and canopy chlorophyll content (CCC) of potato plants over an agricultural field for six dates during the growing season. We analyzed: (1) The standard look-up table method (LUTstd), (2) an improved (regularized) LUT method that involves variable correlation (LUTreg), (3) hybrid methods, and (4) random forest regression without (RF) and with (RFexp) the exposure time as an additional explanatory variable. The Soil–Leaf–Canopy (SLC) model was used in association with the LUT-based inversion and hybrid methods, while the statistical modelling methods (RF and RFexp) relied entirely on in situ data. The results revealed that RFexp was the best-performing method, yielding the highest accuracies, in terms of the normalized root mean square error (NRMSE), for LAI (5.36%), fCover (5.87%), and CCC (15.01%). RFexp was able to reduce the effects of illumination variability and cloud shadows. LUTreg outperformed the other two retrieval methods (hybrid methods and LUTstd), with an NRMSE of 9.18% for LAI, 10.46% for fCover, and 12.16% for CCC. Conversely, LUTreg led to lower accuracies than those derived from RF for LAI (5.51%) and for fCover (6.23%), but not for CCC (16.21%). Therefore, the machine learning approaches—in particular, RF—appear to be the most promising retrieval methods for application to UAV-based hyperspectral data.

## 1 Introduction

Crop trait assessment and monitoring are of crucial importance in agricultural applications (i.e., precision farming) [1]. By providing an accurate estimation of crop traits, the accuracy of growth monitoring can be improved. Consequently, spatially explicit trait quantification can help a farmer to adapt and optimize their management practices (e.g., nutrient application) in an efficient way, in order to increase the yield production [2]. The leaf area index (LAI) and fractional vegetation cover (fCover) are key canopy structural variables, used for characterizing the ecological, hydrological, and biogeochemical processes in terrestrial climate systems [3]. Furthermore, chlorophyll content, defined either at the leaf level (leaf chlorophyll content, LCC) or at the canopy level (canopy chlorophyll content, CCC) is used as a bioindicator of vegetation state [4–6]. These variables of interest are also good proxies of above-ground biomass, nitrogen uptake, and the actual crop condition [7,8].

Remote sensing observations are valuable data sources for mapping the spatial variation of crop traits in precision agriculture applications [9]. In particular, the possibility to acquire hyperspectral data at a very high spatial resolution repeatedly during a crop season and the whole plant phenology has led to the creation of new applications for drone- or UAV (unmanned aerial vehicle)-based observations [10,11]. Since the new technology of UAV-based hyperspectral data has appeared, several studies have been devoted to obtaining good predictions of LAI [1,12–17], fCover [18–20], and CCC [21–23].

Over the last few decades, a diversity of retrieval methods has been developed, enabling the conversion of reflectance data into certain variables of interest. These models can be broadly classified into three general categories: Physically based, statistical, and hybrid methods. The physically based methods (i.e., radiative transfer models; RTMs), are considered generic, transferable, and independent of in situ measurements [24–26]. However, the ill-posed problem has been encountered as one major limitation for RTM inversion strategies, as several combinations of input variables may result in identical spectra [27]. Moreover, measurement uncertainties and model assumptions may induce a large variation of possible solutions, leading to inaccurate results for the estimated variables [28]. To alleviate the ill-posed inverse problem and increase the accuracy of retrieval, different regularization schemes have been suggested, as described in [29]. In a recent study [30], the usage of correlated variables using the Cholesky method was proposed, in order to regularize a look-up table inversion approach (LUTreg). Using *prior* information on the cross-correlations between variables (e.g., LAI, CCC, and fCover), which can be collected from field measurements, may reduce the probability of unrealistic parameter combinations and simulated spectra [31].

Progressing along this line, a comparison of the LUTreg method with other retrieval methods is needed. Statistical methods involve parametric and non-parametric regression methods [32,33]. Machine learning algorithms (ML), as non-parametric regression methods, often markedly outperform parametric methods, since the relationships between crop variables and the observed reflectance often entail non-linear variability and autocorrelation [34,35]. Due to its dependence on the ground data, however, a statistical method may be poorly transferable to other sites, vegetation types, or sensors [24]. Furthermore, its performance may be hampered by the number, quality, and representativeness of in situ data [36]. Nevertheless, such methods are of interest to researchers, due to their flexibility in predicting the variable of interest [37,38].

The last category of retrieval methods is hybrid methods, which use ML algorithms for training spectra simulated by an RTM. Hybrid methods appear promising, as they combine the universality and robustness of physical models with the advantages of non-parametric methods (e.g., non-linearity, fast performance) [39–42]. To train the generated LUT database, a variety of ML algorithms have been introduced into hybrid methods for retrieving canopy traits. Among ML algorithms, Random Forest Regression (RF) and Gaussian Process Regression (GPR) have been well applied in several studies, due to their robustness and efficient implementation [39,43–49]. RF is a regression tree-based ensemble algorithm which can handle several input variables without overfitting while also being less sensitive to outliers and noise [50,51]. GPR was developed based on the theory of the Bayesian framework. Fortunately, it does not require a large sample size for the training data set and needs less tuning for the hyperparameters [52]. Additionally, the uncertainty of estimates can be provided by calculating the standard deviation and mean [53]. As compared to GPR and RF, Conical Correlation Forest (CCF) has received less attention in retrieval studies [54]. The sensitivity of this method to the ensemble size (i.e., the number of trees) is less than that of RF [55]. Therefore, it is worthwhile to evaluate alongside other methods (e.g., RF and GPR).

A specific challenge arises when UAV-based hyperspectral data are acquired under sub-optimal illumination conditions, a condition that is quite abundant in, for instance, Central and Northern Europe, featuring a mixture of full or partial cloud cover and clear sky during a growing season. Indeed, processing such images taken under variable illumination throughout the flight mission and the growing season of the crop is not straightforward. In the studies of [56,57], additional radiometric calibration beside the empirical line method has been used to reduce this effect, based on irradiance measurements by ASD FieldSpec3 or UAV and the integrated exposure time. However, when this information is not available, UAV images taken under cloudy conditions are mostly discarded [58–60]. To the best of our knowledge, no previous study has been devoted to systematically assessing how the accuracy of variable estimates using the aforementioned methods is affected while operating a UAV under variable illumination conditions (i.e., cloudy and partially cloudy weather).

Overall, this study set out to investigate the performances of statistical, physical, and hybrid methods under variable illumination for estimating LAI, fCover, and CCC from UAV-based hyperspectral data throughout the growth cycle of a potato crop. The following specific objectives are addressed: (1) To test whether the regularized LUT (LUTreg) developed in the study of Abdelbaki et al. [30], which was shown to work on a single observation date, using field spectroradiometer measurements, can yield an improvement in the variables of interest; (2) to compare the LUTreg with statistical and hybrid methods in the estimation process; and (3) to assess if using information on illumination conditions during image acquisition can improve the accuracy of estimates.

## 2 Materials

### 2.1 Study Area and Experimental Setup

**Figure 1:**
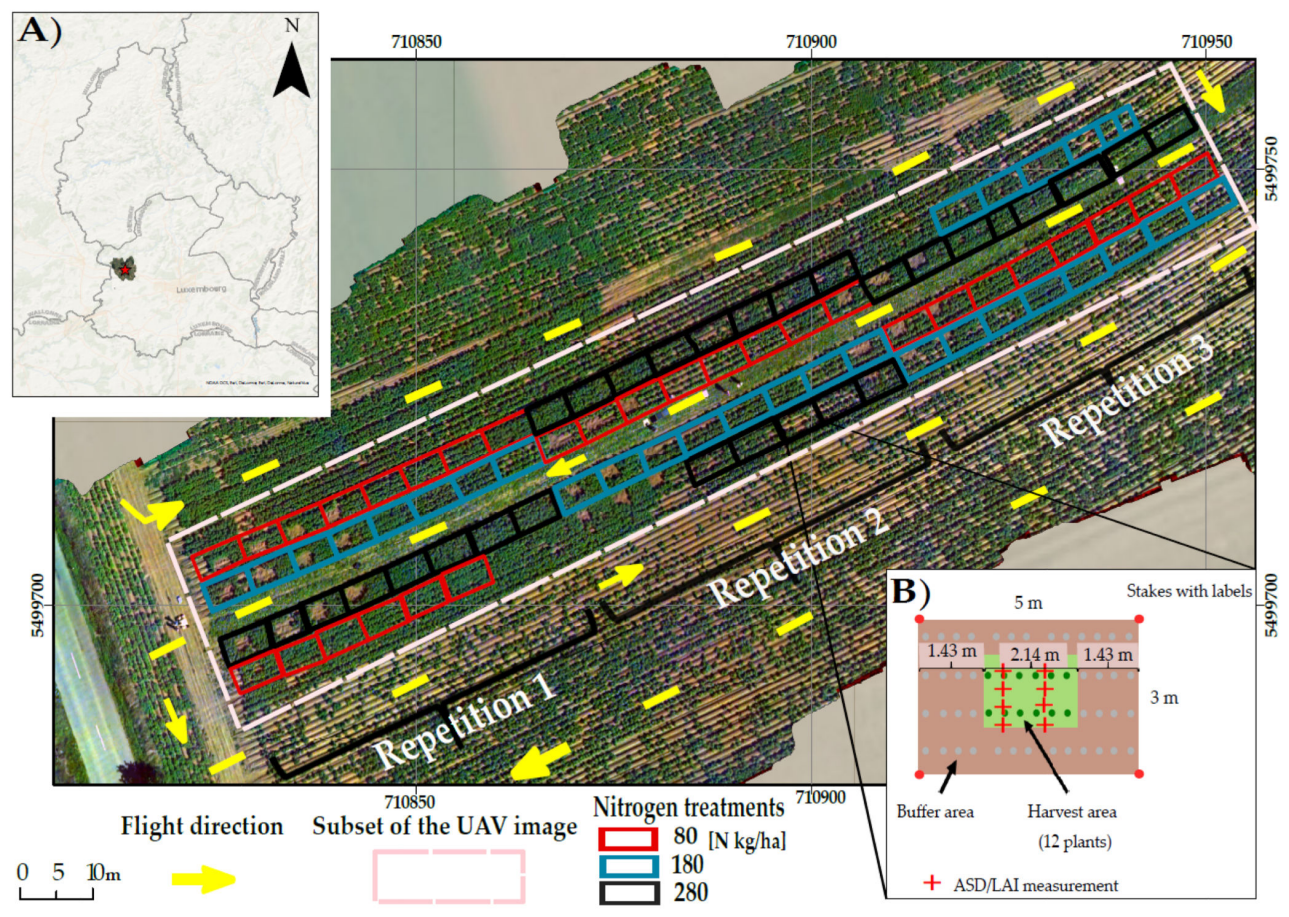
(**A**) A map of Luxembourg, containing the exact location of the study site (the red star). Main map: Image of the UAV orthomosaic, based on the true RGB color captured by the drone on 19 July at an altitude of 50 meters (12:15 pm); and (**B**) the plot design for in situ measurements [30].

The study area is in the southwest of Luxembourg, close to Hivinge village 49°36′47.1″N, 5°55′6.7″E (Figure 1A). A potato cultivar (*Solanum tuberosum* L. cv. Victoria) was cultivated in the spring/summer season of 2016. The predominant soil type was sandy loams. The annual mean temperature and annual total precipitation of the study area were 9.8 °C and 865 mm, respectively. Six field sampling dates for the potato crop were conducted, from 8 July to 10 August, resulting in 156 sampling plots (see Table 1). Plots (5 m × 3 m) with three different levels of nitrogen application were established (80, 180, and 280 kg/ha nitrogen), representing under-, standard-, and over-fertilization, respectively (see Figure 1B), which led to variation in biophysical and biochemical variables. Each fertilization level was represented by three replicates times three plots (for nine samples), leading to 27 samples at each observation date. However, on the first date of the growing seasons, there were only 21 valid samples, as the flight did not cover the whole field by mistake.

Non-destructive measurements were taken in the center area of each plot, along with the positions (Figure 1B), in order to avoid border effects. To determine the non-destructive LAI from the gap fraction an LAI-2000 Plant Canopy Analyzer (PCA; Li-Cor, Inc., Lincoln, NE, USA) was used in this study. The optics of PCA consist of a fisheye lens (148° field of view (FOV)), which encompasses five sensors, each si-simultaneously measuring light intensities in the blue spectral-domain (320–490 nm) (with central zenith angle of 7°, 23°, 38°, 53°, and 68°, respectively) [61,62]. A 180° view cap of the PCA lens was fixed and the PCA measurements were processed to LAI by the File Viewer software (LI-COR FV2000). The measurements were taken either early in the morning, under clear-sky or partly cloudy conditions, or near mid-day, during overcast sky conditions, to minimize the effects of direct radiation. In each plot, below-canopy readings were recorded at eight different positions within the plot (red crosses in Figure 1B), followed by an above-canopy reading. fCover was visually estimated by an experienced observer, in a vertical plant shoot-area projection, as a percentage of quadrat area, where the observer divided the range of fCover (0–100%) into interval classes (in steps of 5%) as an ordinal variable [63]. The leaf chlorophyll content (LCC) was measured using an SPAD-502 Konica Minolta. Six leaves per plot were selected randomly, and the five readings per leaf from different positions of the top leaflet were averaged to one value. The SPAD readings (unitless) were converted into LCC (μg cm^−2^). The Formula (1) developed by [64] was applied for unit conversion. The CCC (g cm^−2^) for each plot was then determined, using multiplication between LAI and LCC values at leaf level.

(1) $$LCC\left(\mu \text{g}\;{\text{cm}}^{-2}\right)=0.0913*{\text{e}}^{0.0415*SPAD}.$$

### 2.2 Canopy Spectra Measurements

Spectral measurements of each plot were performed using an ASD FieldSpec3 spectroradiometer (Analytical SpectralDevices, Boulder, CO, USA) at six critical growth stages with a spectral range from 300–2500 nm. The spectral resolution of 2151 spectral bands was 3 nm between 350–1000 nm and 10 nm between 1000–2500 nm. Before carrying out the UAV flight missions, ASD canopy measurements were taken at nadir position under direct solar illumination between 10:30 a.m. and 2:00 p.m. (one hour before and after on the same day of the flight mission), from a distance of about 80 cm above the canopy. With a field of view of 25°, a footprint diameter of 0.88 cm^2^ on the ground was observed. For each plot, the eight measurements at distributed positions (red crosses; Figure 1B) were averaged.

### 2.3 UAV-Based Hyperspectral Data Acquisition and Processing

Prior to the hyperspectral flight missions, a fixed-wing aircraft UAV (Mavinci Sirius) equipped with a Real-Time Kinematic Global Positioning System (RTK-GPS) was flown over the larger study site, in order to obtain a reference orthomosaic of the area, on 22 June 2016. The UAV contained an RGB-camera. The captured images were processed to an orthomosaic, using the Agisoft Photoscan Processional software (v. 1.26 Agisoft, LLC, St. Petersburg, Russia), to a final ground resolution of about 1.7 cm and registered to the local Universal Transverse Mercator Zone 31 North projection based on the World Geodetic System (UTM 31N WGS 1984).

Aerial images for the experiment were acquired during six flight missions in 2016 (Table 1) using an OXI VNIR-40 hyperspectral sensor (Gamaya, SA, Lausanne, Switzerland) system, which was mounted on a DJI S900 octocopter UAV. The sensor system contains two global snapshot cameras with a 5 × 5 grid sensor for 25 spectral bands in the visible range (VIS) between 474 nm to 638 nm (FWHM of 16–27 nm), as well as a 4 × 4 grid sensor for 16 spectral bands in the near-infrared between 638 nm to 915 nm (FWHM of 15–27 nm), with partly overlapping bands. The raw VIS and NIR images were de-convolved by Gamaya, resulting in two images with size of 2048 × 1088 pixels (2 MP). Using the Agisoft Photoscan Professional software (v. 1.26, Agisoft, LLC, St. Petersburg, Russia), the two sets of images (VIS and NIR) were further processed by Gamaya. The focal length of the camera was 25 mm and the exposure time was set manually, prior to each flight. The duration of flight missions was 15 min. The aerial images were captured at an altitude of about 50 m, with a front lap of 75% and a side lap of 60%. As GPS positions were not automatically stored during image acquisition, all orthomosaic images were co-registered to the RTK-RGB-orthomosaic as the base reference, with an RMSE_xy_ of 0.78 to 0.14 pixels.

In our campaigns, UAV data acquisition was carried out bi-weekly and deliberately under less favorable illumination conditions (Table 1). For radiometric calibration to reflectances, nine wooden panels with grey shades from black to white were laid out at the center of the study area. Their radiance was measured during the image acquisitions and calibrated to reflectances. The optimal illumination date, 19 July 2016, was selected as a radiometric reference (Figure 1A). An empirical line calibration (ELC) [65] was carried out between the optimal illumination date (19 July) digital numbers (DN) and the reference panel reflectances obtained from the ASD FieldSpec3 measurements in the field on that date. Due to saturation of the brightest panels, only the five darkest panels were integrated into the derivation of the ELC for the VIS sensor. However, for the NIR sensor, all reference panels were used. All values were scaled from 0–1. The empirical line (EL) fit was evaluated using R^2^ and the residuals (RMSE) (Table A1). All hyperspectral orthomosaics for the other dates were calibrated to the target reflectances of the reference panels measured by the ASD FieldSpec3 in the field on the reference date (19 July). Table A1 lists the radiometric accuracy for each date’s first 40 bands. The 41st band was eliminated, as it contained a sharp drop in reflectances, which was not explicable. The mean values for the six flight dates range from R^2^ = 0.99 (RMSE = 0.01) for the reference date (19 July) to R^2^ = 0.94 (RMSE = 0.04) for the following flight date (27 July), where the lightning conditions varied the most, indicating mean reflectance errors below 2% vs. 5% for the worst case.

**Figure 2:**
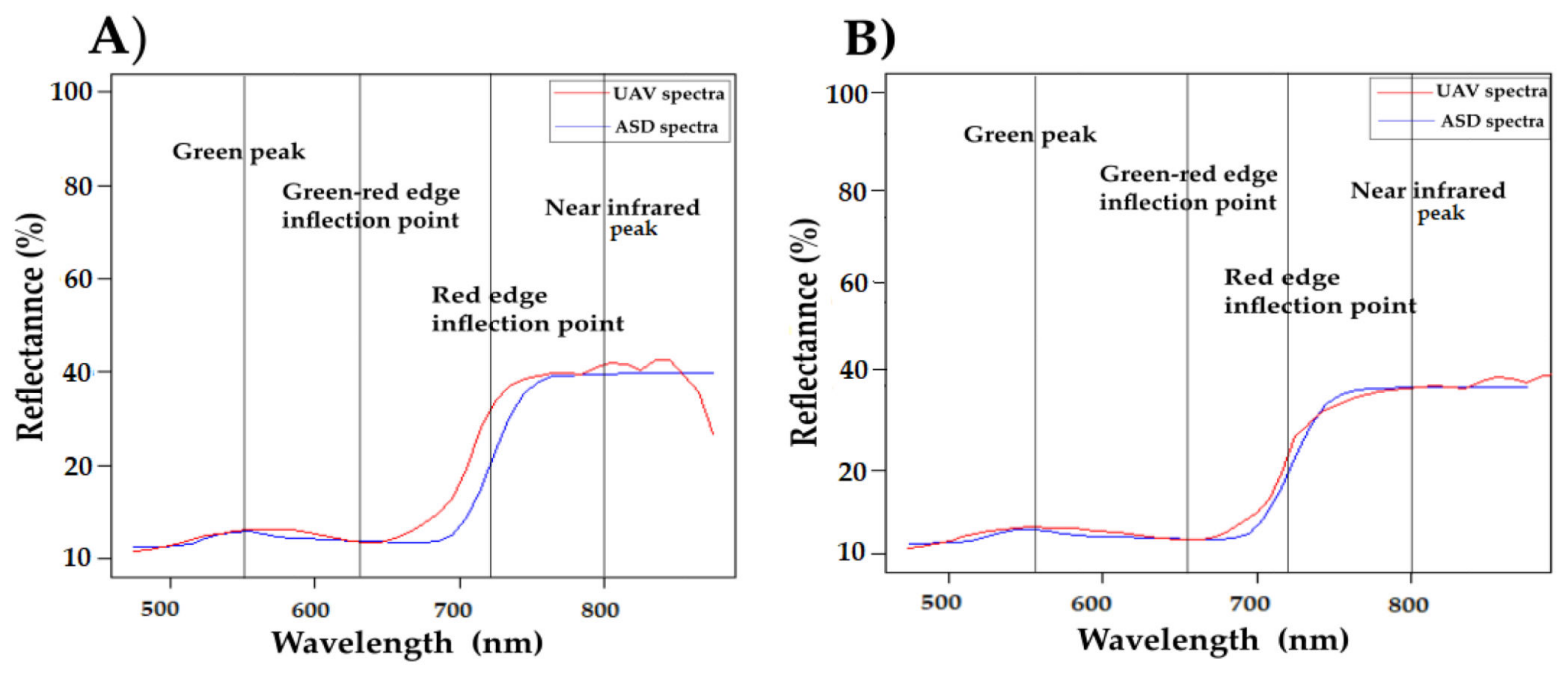
Comparison of the canopy reflectance between spectra derived from a field spectrometer (ASD FieldSpec3, blue) and UAV spectra (red) before (**A**) and after (**B**) correction of spectral band positions for a representative plot (LAI = 2.04 m^2^/m^2^, fCover = 0.65, and CCC = 1.44 g/m^2^) on 19 July.

When comparing the canopy reflectances collected in situ to the spectra obtained by the OXI VNIR-40 sensor, spectral shifts between distinct spectral features were detected due to a broad bandwidth of the spectral bands of Gamaya-camera. The resulting overlap of individual spectral bands results in spectral smoothing and a related shift in spectral band positions. This is comparable to a low-pass filtering effect, as shown in Figure 2A. Therefore, a systematic test was implemented based on a comparison between the ASD FieldSpec3 and UAV image sample spectra of the identical plot area. Characteristic spectral shape features, such as maximum peaks of green and NIR, the inflections points of the green-red edge, and the red edge inflection point, were used by means of first and second derivative of the hyperspectral reflectance curve. The OXI sensor band positions were then shifted towards the ASD band positions by spline interpolation, showing the result of the OXI spectrum in the red curve (Figure 2B).

## 3 Methods

### 3.1 Radiative Transfer Model

The Soil–Leaf–Canopy (SLC) model [66], a combination of the leaf model PROSPECT-4 [67], the canopy model 4SAIL2 [66], and the soil model Hapke [68] were used in this study to predict the LAI, CCC, and fCover of potato crops (heterogeneous and discontinuous crop). The SLC model does not have many input parameters to optimize, as compared to other complex 3D models (e.g., DART [69]), which are mainly used when considering a spatially heterogeneous plant canopy [70]. Moreover, the fCover variable is directly quantified as a model output, compared to other RTMs (e.g., PROSAIL [71]).

SLC simulates canopy reflectance over the spectral range between 400 and 2500 nm with a spectral resolution of 1 nm. The PROSPECT-4 model simulates directional-hemispherical reflectance and transmittance for a single leaf. The input variables of the model are the leaf structure parameter (N) and leaf biochemical constituents, including leaf chlorophyll content (LCC), leaf dry matter content (Cm), leaf water content (Cw), and leaf senescent matter content (Cs). The 4SAIL2 model, which is an amended version of the turbid medium SAIL model, simulates the top of the canopy reflectance. This model is a function of a series of variables: The fraction of brown canopy area (fB), the dissociation factor (D), hotspot (hot), tree shape factor (Zeta), crown cover (Cv), leaf area index (LAI), and leaf inclination distribution function (LIDF a and b). The latter three input variables were used to retrieve the effective fraction of vegetation cover (fCover) [72], as follows:

(2) $$fCover=Cv*\left(1-e^{-k*LAI}\right),$$

where *Cv* is the vertical crown cover, e^–k*LAI^ is the gap fraction following the Lambert–Beer law, and *k* is the extinction coefficient in the vertical direction, which depends on the leaf inclination distribution function (LIDF) and the viewing angle (Θ).

Moreover, in the Hapke model, the soil moisture content (SM) and Hapke parameters are employed for simulating soil spectra (bidirectional reflectance distribution function; BRDF). To observe the geometry of the UAV image capture, the observed zenith angle (tto), relative azimuth angles (psi), and the solar zenith (tts) (28°, 29°, 30°, 31°, 33°, and 35°) parameters were fixed at nadir-viewing position under different field conditions. To constrain the inversion result, the ranges and distributions of free variables (LAI, LCC, and Cv) were set, based on *prior* knowledge from ground measurements, while the remaining parameters were fixed based on the literature, as shown in Table 2.

### 3.2 Look-Up Table Generation-Based SLC Model

Extending a previous study [30], we generated two LUTs (LUTstd and LUTreg) with a size of 17,280 simulations. The input model variables of the standard LUT (LUTstd) were independent of each other, following the uniform and multivariate normal distribution function. To cover and maximize the sampling space of input variables, Latin Hypercube Sampling (LHS) was employed [73]. On the other hand, the regularized LUT (LUTreg) relied on the correlated variables naturally found in the field. There were strong correlations between measured LAI and fCover (R = 0.83), LAI and CCC (R = 0.97), and CCC and fCover (R = 0.79) variables. To preserve their relationships and law distributions in the SLC model (Figure A1 and Table 2), the Cholesky method (LU) combined with Latin Hypercube Sampling (LHS) was used to create the cross-correlation between the model input variables of LAI, Cv, and LCC [74,75].

The detailed calculations of the proposed algorithm used in LUTreg are described as follows, broken down into steps and implemented using Matlab 2019:

(1) Initialize the number of canopy simulations (n = 17,280) and the number of correlated variables with their normal distributions.
(2) Generate the Latin Hypercube Samples (Z) with the size of n × 3, considering the number of canopy simulations (n) and correlated variables (3) that divide into samples (N), with the same probability of 1/N, and selecting one sampling value of these samples in each partition randomly [76].
(3) Define the correlation matrices between three measured variables (M) and between the generated values of LHS (*m*), following the size of the correlated variables.
(4) Calculate the non-singular lower triangular matrix (*L*) of the measured variables by using the Cholesky decomposition method (LU) for the correlation or covariance matrix (*M*), which satisfies:
  (3) $$M=LL^{T}=\left[\begin{array}{ccc} 1 & R & R\\ R & 1 & R\\ R & R & 1 \end{array}\right]=\left[\begin{array}{ccc} \sigma_{i} & 0 & 0\\ a & b & 0\\ c & d & e \end{array}\right]\left[\begin{array}{ccc} \sigma_{i} & a & c\\ 0 & b & d\\ 0 & 0 & e \end{array}\right]=\left[\begin{array}{ccc} \sigma_{1}^{2} & \rho_{1,2}\sigma_{1}\sigma_{2} & \rho_{1,3}\sigma_{1}\sigma_{3}\\ \rho_{1,2}\sigma_{1}\sigma_{2} & \sigma_{2}^{2} & \rho_{2,3}\sigma_{2}\sigma_{3}\\ \rho_{1,3}\sigma_{1}\sigma_{3} & \rho 2,3\sigma_{2}\sigma_{3} & \sigma_{3}^{2} \end{array}\right],$$
  (4) L=Cholesky(M),
  where *M_(3 × 3)_* is a Hermitian positive-definite matrix, which is decomposed into lower triangular (*L*) and upper triangular (*L^T^*) matrices; *R* is the correlation coefficient used in the correlation matrix M; *σ_i_* is the standard deviation of the variable *x_i_*; *ρ*_i,j_ is the covariance between *x*_i_ and _j_; *a*, *c*, and *d* could be positive, negative or zero values of off-diagonal values; and *b* and *e* cannot be equal to zero.
(5) Calculate the non-singular lower triangular matrix (*Q*) from the correlation matrix of the LHS realizations (*m_(3 × 3)_*):
  (5) Q=Cholesky(m).
(6) Simulate the correlated random variate, which is based on transforming the realization matrix of LHS (*Z*) to a new matrix, denoted *Z*_1_, with size n × 3.
  (6) $$Z_{1(\text{i},\text{j},\text{k})}=Z_{\left(\text{n}\times 3\right)}*{\left(L_{\left(3\times 3\right)}*Q_{\left(3\times 3\right)}\right)}^{T}.$$
(7) Convert the uniform correlated variables of *Z*_1_ to the normal distribution function, as defined before for three variables (Step 1). Then, each product of Z_1_ represents (Z_1i_) for LAI, (Z_1j_) for Cv, and (Z_1k_) for LCC.

More details about the implementation of the Cholesky method (LU) and LHS to correlate the model inputs using the ground measurements can be found in [30,76]. The simulated spectra of both LUTs (LUTstd and LUTreg) were resampled, corresponding to the 40 bands of the Gamaya OXI VNIR-40. To reduce the uncertainties of the modelled and measured spectra, as well as autocorrelation between the spectrum and input variables, a Gaussian distribution with 0.5% was injected into the canopy simulations [77].

As the fully green leaves of the potato crop were observed during the growing season, the value of the fraction of the brown canopy area (fB) was set to zero (Table 2). Furthermore, the value of the leaf senescent material (Cs) was fixed as zero, using our knowledge in the field. To characterize the mixture between green and brown leaves, the D parameter is set equal to zero when brown leaves are homogeneously distributed over the top layer of the canopy. On the other hand, when the brown leaves are at the bottom of the canopy layer, the D value is equal to 1, as was observed in our field trial. The tree shape factor (Zeta) was calculated based on the ratio of crown diameter to crown height. The values of crop height and crown diameter were roughly set as 100 cm, such that the value of zeta was defined as 1. The range of Cv values used in 4SAIL2 was defined according to the study of [30], based on Equation (2) with a fixed value of the extinction coefficient (K = 0.55). Lastly, the Hapke parameters were set as default values for ploughed soil [66], due to a lack of information in the soil measurements.

### 3.3 Retrieval Strategies

#### 3.3.1 Physically Based Method

The retrievals from LUTstd- and LUTreg-based inversion were applied to 156 of the measured spectra recorded during six observation dates and at three levels of nitrogen fertilizers (N80, N180, and N280). The RMSE was used as a cost function, in order to find the closest match between measured and simulated spectra, sorted in ascending order (i.e., lowest to highest). Consequently, the best single solution for a possible variable combination could be defined. However, this solution may not be the unique or optimal result, due to measurement errors and model inadequacies [25]. In an attempt to solve the inverse problem, averaging similar input variable combinations with the smallest differences between the measured and simulated spectra were calculated using the mean or median [25,81,82]. However, the median of 300 LUT entries was used as an optimal solution, as has been pointed out previously [30].

#### 3.3.2 Hybrid Method

From the available ML algorithms in the MLRA toolbox of ARTMO, three approaches were selected for this study (Table 3). They were classified into non-kernel and kernel regression approaches: Random forests (Tree Bagger; RF) and Conical correlation forest (CCF) as non-kernel methods; and Gaussian process regression (GPR) as a kernel method. After identification of the best type of inverted LUTs (LUTstd and LUTreg), the optimal LUT—containing the pairs of modelled spectra and corresponding input model variables—was used for training. Using a large training data set of simulations may not be beneficial in several ML algorithms, due to the redundant information and the calculation time [83]. To reduce the size of the training sets, different subsets, starting from 100 to 5000 simulated spectra, were randomly sampled from the original pooled data set (17,280 simulations). The ten subsets were used for training and testing the three ML methods. This procedure was repeated ten times, using the k-fold cross-validation strategy. In order to identify the most appropriate sample size and method of ML, ground validation based on in situ data, as an independent test, was mainly used for evaluation; instead of using the cross-validation relying on the simulated data set from the SLC model (i.e., theoretical validation).

#### 3.3.3 Statistical Method Using the Exposure Time

Among the ML methods presented in Table 3, Random forest regression (RF) was selected to train the in situ data. RF provides high accuracy for estimation without a tendency for overfitting [39,42,84]. Thus, it enabled us to evaluate the performances of LUTreg and hybrid methods, where no in situ data were used for the model calibration.

With the aim of reducing the effect of spatial autocorrelation in the 156 experimental samples, leaving-one-sampling-date-out (n = 27) as unseen data in the model was performed. This procedure was repeated for each sampling date, except for the first date, in which the model was trained with 135 samples and the remaining samples (n = 21) were used for validation. As image acquisition in our experiment was carried out under varying illumination conditions, the exposure time was added as independent variable besides the measured spectra to estimate LAI, fCover and CCC variables during the training process (hereafter denoted as RFexp).

### 3.4 Model Validation

The LAI, fCover, and CCC estimates obtained from the retrieval methods were assessed using common statistical indicators, such as the coefficient of determination (R^2^), normalized root mean square error (NRMSE (%), and root mean square error (RMSE) (Equation (3)). In hybrid methods based on ML, the Friedman test [88], followed by a pairwise multiple comparison test using the Bergmann–Hommel procedure adjusted for *p-values*, was performed to determine whether one of the MLRAs was statistically significant in the estimates. For the LUT inversion methods (LUTreg and LUTstd), the paired t-test was applied to evaluate the significance between different levels of nitrogen.

(7) $$NRMSE\%=\frac{RMSE}{\text{range}\;\text{of}\;\text{measured}\;\text{variable}}*100.$$

After evaluating the accuracy assessment between retrieval strategies, the best method was used to map the canopy traits using UAV images taken under sunny conditions (19 July). The non-potato crop area (soil and weeds) was manually masked out from the UAV image using the ENVI software (v. 5.1, Harris Corporation, Melbourne, FL, USA). Then, the best retrieval method was applied to the potato crop-masked images.

## 4 Results

### 4.1 Descriptive Statistics of Field Measurements

Table 4 shows the detailed summary statistics of the biochemical and biophysical characteristics (LAI, fCover, and CCC) for 156 potato samples. The table reveals that the mean of measured variables increased continuously, until they reached a maximum value on 5 August (maturity stage). At the end of the growing season (10 August), the mean of measured variables decreased, when senescence took place. Furthermore, based on the combined data of the six observation dates, there were high variabilities in the coefficient of variation (C.V.) measures for LAI and CCC variables, as compared to fCover. This indicates that the measured fCover was more stable than that of measured LAI and CCC.

### 4.2 LUTreg- and LUTstd-Based Inversion

**Figure 3:**
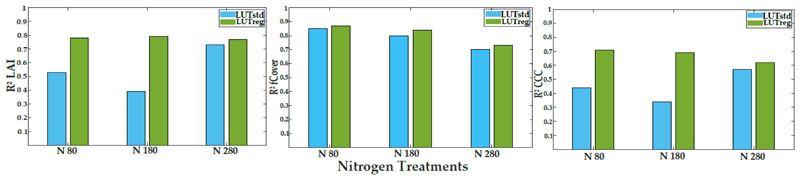
The coefficient of determination (R^2^) obtained from LUTreg and LUTstd in predictions of LAI, fCover, and CCC at three levels of nitrogen.

**Figure 4:**
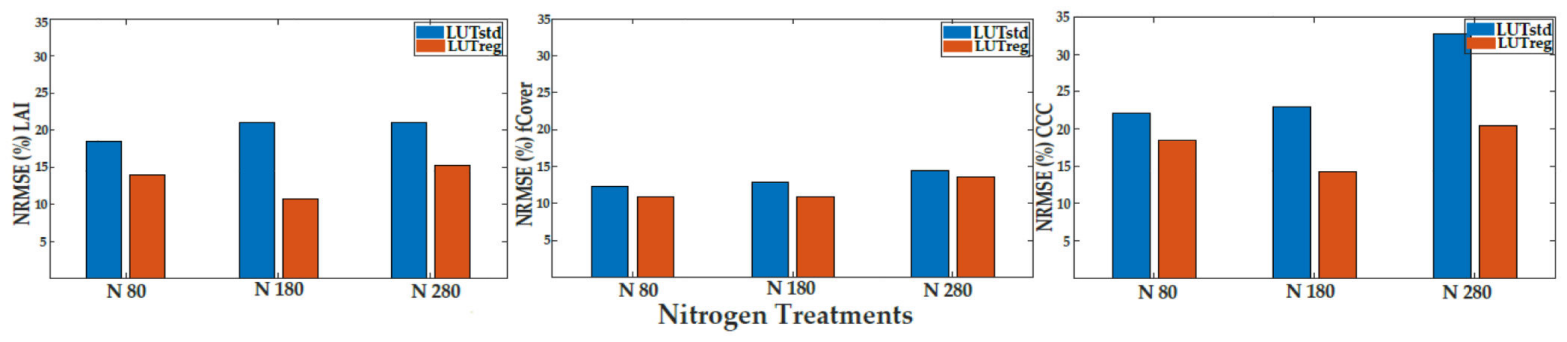
The normalized root mean square error (NRMSE%) obtained from LUTreg and LUTstd in predictions of LAI, fCover, and CCC at three levels of nitrogen.

The performance of LUTreg was evaluated against LUTstd, in terms of LAI, fCover, and CCC predictions, using the whole data of the six observation dates. At different levels of nitrogen fertilization, there was a clear enhancement in LAI and CCC estimates, but not for the fCover estimate (Figures 3 and 4). With the treatment rates of nitrogen (N80 and N180), the accuracy, in terms of R^2^ and NRMSE (%), improved in the estimated LAI and CCC; however, when increasing the level of nitrogen (N280), their accuracies started to decrease. For fCover, the observation was the same as LAI and CCC, where the accuracy improved slightly in LUTreg with the standard level of nitrogen (N180).

**Figure 5:**
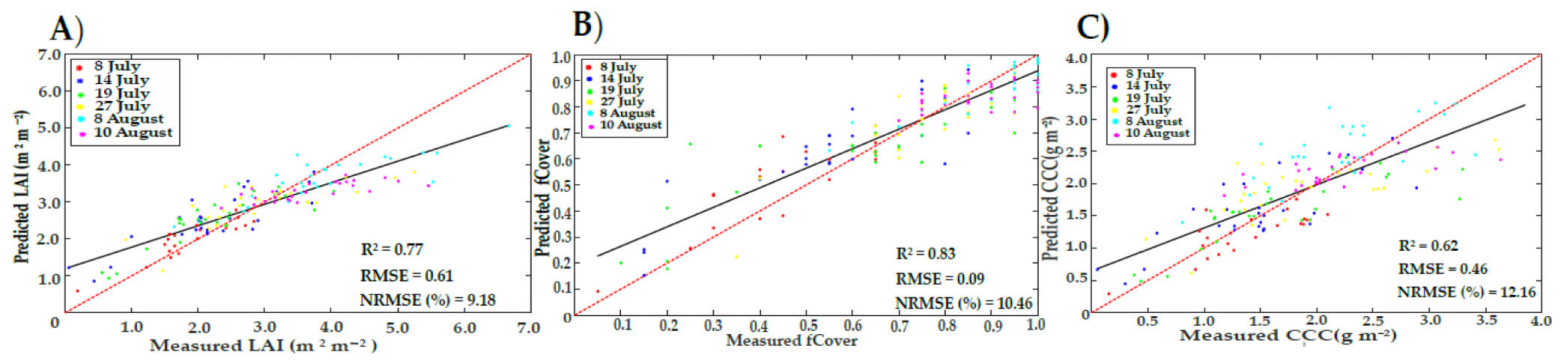
Scatterplots of LAI (**A**), fCover (**B**), and CCC (**C**) obtained from LUTreg using all data (156 samples); with trend lines for linear fitting (black) and 1:1 line (dashed red).

Using the paired t-test, significant differences between the two types of LUTs (LUTstd and LUTrteg) were observed for LAI, fCover, and CCC, at *p* < 0.05. Significant differences between the three levels of nitrogen in the estimations were also found between LUTreg and LUTstd. Figure 5 represents the tendency of predictions to assess the permanence of LUTreg and LUTstd, in terms of over-or underestimation. LUTreg underestimated the values of LAI (above 4), fCover (above 0.8), and CCC (above 2.5). However, in LUTstd the underestimation of LAI, CCC, and fCover started above 3, 1.5, and 0.8 values, respectively (Figure A2).

### 4.3 Hybrid Methods Based on ML

The validation accuracies of the predicted LAI, fCover, and CCC by the three selected ML methods are presented to identify the best method (Table 5) and best sample size using all data sets (n = 156 samples), as shown in Appendix A and (Table A2 (for LAI), Table A3 (for fCover), and Table A4 (for CCC). GPR outperformed the other methods, when using a sampling size of 100 samples, for LAI (R^2^ = 0.70, NRMSE% = 9.80). RF and CCF were the best methods for fCover and CCC when using sample sizes of 500 and 1000, respectively (R^2^ = 0.82 and NRMSE% = 10.59 for fCover; and R^2^ = 0.55 and NRMSE% = 13.4 for CCC).

**Figure 6:**
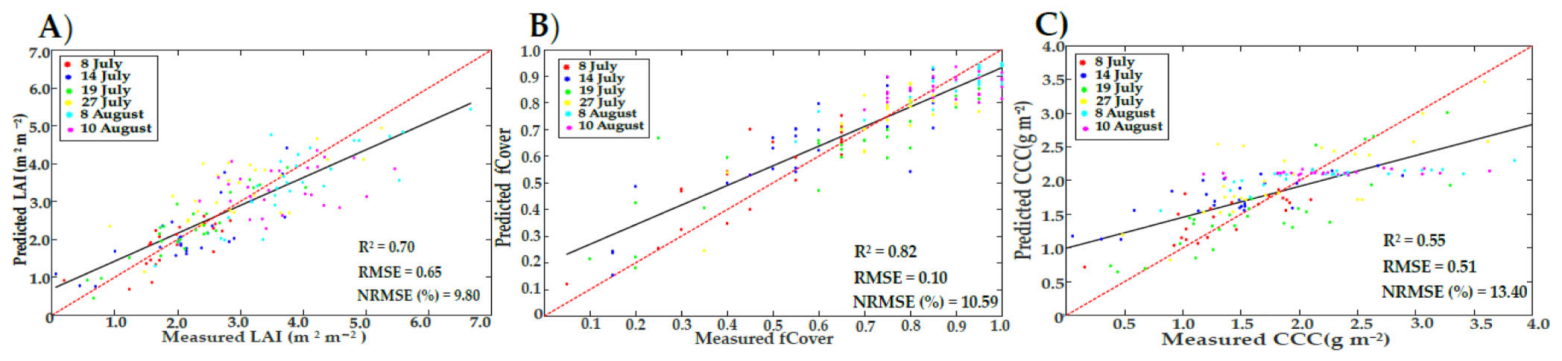
Scatterplots of LAI, fCover, and CCC predicted from hybrid models based on GPR (**A**), RF (**B**), and CCF (**C**), respectively; with trend lines for linear fitting (black) and 1:1 line (dashed red).

There were significant differences between the three hybrid methods (p < 0.05) for LAI (0.0045), fCover (0.00063), and CCC (0.000018). When assessing the significant difference between LUTreg and the best method of ML, LAI did not show any difference (0.082), as compared to fCover (0.00013) and CCC (0.0000029). Scatter plots (Figure 6) show that the estimated CCC had a strong tendency of underestimation for higher values (above 2.5), as compared to LUTreg. For other estimated values (LAI and fCover) using GPR and RF, the underestimation phenomena appeared the same as those obtained with the LUTreg method.

### 4.4 Retrieval Strategies Under Illumination Variation and Crop Developments Over Time

The results presented in Table 6 summarize the effects of illumination variability and growth stage on the estimation accuracies when using different retrieval methods. In general, RFexp delivered high accuracies for LAI, fCover, and CCC through crop development under sunny and cloudy conditions. The four methods were systematically evaluated, based on combining the six observation dates (all data = 156 samples). For LAI and fCover, the rank of retrieval methods, in terms of highest to lowest accuracy, was *RFexp > RF > LUTreg > hybrid;* while the performance of the methods for CCC were ordered as *LUTreg > hybrid > RFexp > RF*.

**Figure 7:**
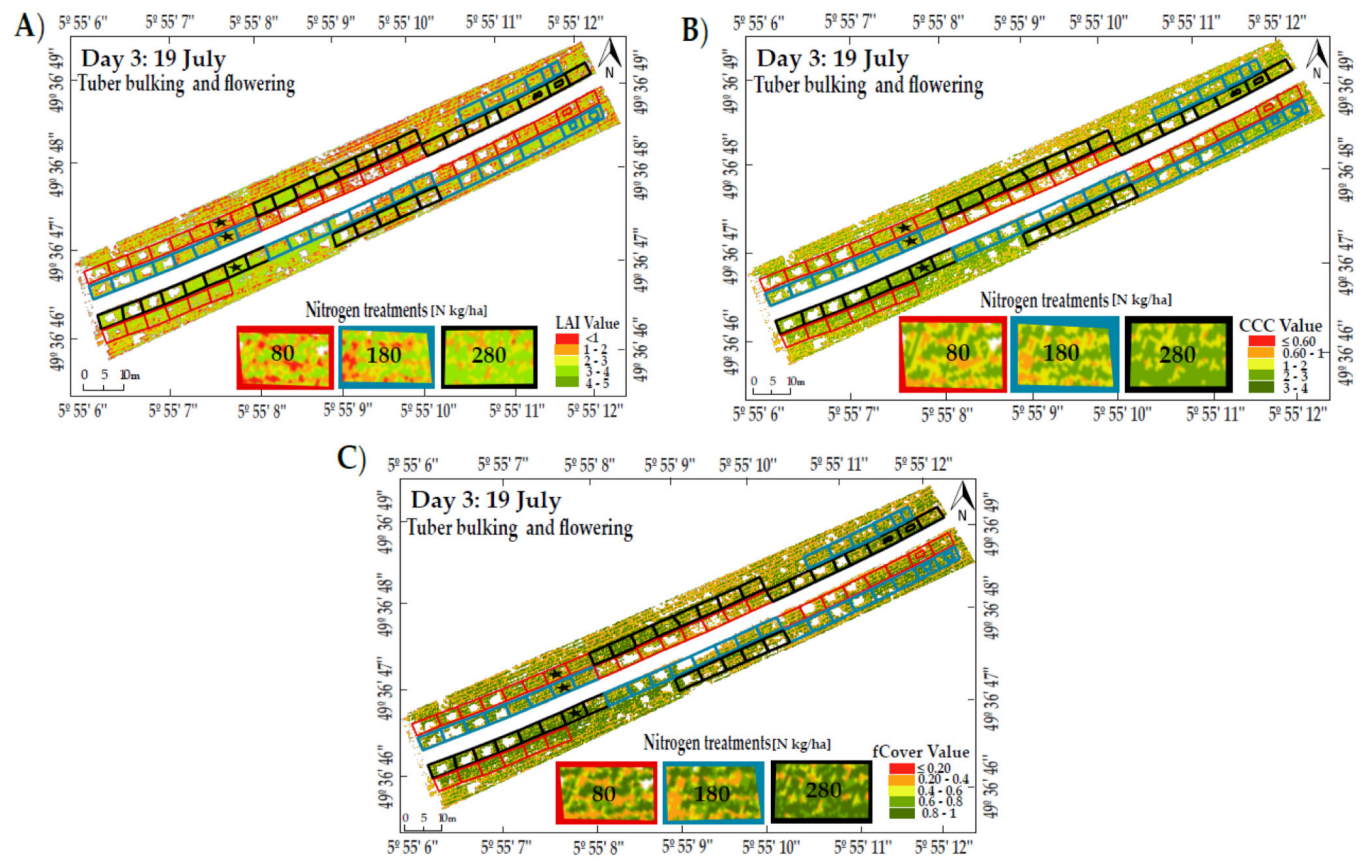
Predictive maps for LAI (**A**), CCC (**B**), and fCover (**C**) using RFexp under sunny day conditions (19 July). The polygons show the applied nitrogen fertilization rates for potato crop development; while the black stars present the exact locations of representative plots.

Variation in illumination obviously impacted LAI retrieval, where sunny days were always the best for statistical methods, as compared to other dates. However, when comparing two methods (LUTreg and hybrid methods) under cloudy conditions and at the maturity stage (5 August), the best accuracy of LAI was obtained from LUTreg. For CCC, the result was inconsistent with LAI using statistical methods (RF and RFexp), where the CCC estimates were not optimal under sunny conditions. Nevertheless, when using hybrid and LUTreg methods, the results of CCC obtained on the date of 5 August were of the same order of magnitude as those obtained for LAI. In the last case for fCover, it was shown that on 27 July (under cloudy conditions), four methods (RFexp, RF, LUTreg, and hybrid) yielded the best accuracy, as compared to other observation dates. A final observation was that on 10 August (the final stage of crop growth), the accuracy of all estimates (LAI, fCover and CCC) started to degrade, which was remarkably consistent with the measured data. Figure 7 shows the spatial distribution of predictive maps for LAI, fCover, and CCC, at different levels of nitrogen, using RFexp for the observation date of 19 July. When increasing the level of nitrogen (N280), the plots displayed more green color (higher values) than the plots under lower levels of nitrogen (N80 and N180).

## 5 Discussion

### 5.1 The Use of Correlated Variables in LUTreg Inversion

In this study, the regularization scheme based on the introduction of variable correlation obtained from the in situ data into LUTreg successfully improved the estimated variables of LAI and CCC at different levels of nitrogen, as compared to fCover (Figure 4). The results from LUTreg emphasized that when increasing the level of nitrogen (N280), the crop cannot respond, leading to a decrease in the accuracy of crop traits (i.e., LAI and CCC). Moreover, the unrealistic simulated canopy spectra in the near-infrared (NIR), which is controlled by several canopy architecture variables (e.g., LAI, LIDF, Cv, Cm, and Cw), decreased, compared to those obtained from LUTstd.

When combining the whole data from the six observation dates (Figure 5), the improvement for estimated fCover (R^2^ = 0.83 and NRMSE =10.46%) was slightly increased in LUTreg, as compared to LUTstd (R^2^ = 0.80 and NRMSE = 13.13%). However, for LAI and CCC obtained from LUTreg, the accuracies were considerably improved (R^2^ = 0.77 and NRMSE = 9.18% for LAI; R^2^ = 0.62 and NRMSE = 12.16% for CCC) rather than LUTstd (R^2^ = 0.61 and NRMSE = 14.45% for LAI; R^2^ = 0.46 and NRMSE = 18.28% for CCC). LUTreg underestimated the high values of LAI (above 4) due to saturation; this result is in line with previous studies [79,89,90]. The SLC model, as an extension of PROSAIL, did not take the row-structure of the potato crop into account; therefore, underestimation often occurred. This, in turn, indirectly affected the CCC estimate; where, with increasing values of CCC (above 2.5), the scattered points were distributed below the 1:1 line. In fCover, underestimation (more than 0.80) took place as the soil background was fully covered by the crop. The assumptions of the SLC model (1D turbid medium RTM) were met, as has been previously reported [30,72].

On the date of 19 July, under sunny conditions, using UAV-based hyperspectral data (Table 6), the accuracy of estimates from LUTreg was R^2^ = 0.73 and NRMSE = 13.87% for LAI, R^2^ = 0.74 and NRMSE = 14.99% for fCover, and R^2^ = 0.64 and NRMSE = 16.11% for CCC. The accuracy of our results was higher than that in previous studies [16,30]. The latter study integrated two correlated variables (LAI and fCover) through the Cholesky method into LUTreg using ASD FieldSpec3 on 19 July. Their findings revealed that the estimated fCover (R^2^ = 0.70 and NRMSE = 17.85%) did not show any improvement, as compared to the estimated LAI and CCC (R^2^ = 0.71 and NRMSE = 25.57% for LAI and R^2^ = 0.70 and NRMSE = 14.01% for CCC). The reason for having a low improvement for estimated fCover in the present study might be that the value of the LIDF parameter was fixed in LUTreg, in order to simplify the model parameterization and avoid confusion with other free variables. The LAI and Cv variables used for quantifying fCover have a great influence on the NIR spectrum and, thus, a problem of the linear spectral mixture of soil and canopy might be introduced in the model simulation for certain plots [30]. Moreover, fixing some parameters, such as leaf dry matter and water content (Cm and Cw), based on the literature, might have had an impact on the final result. The discrepancies between the presented result and the results of [30] on 19 July (sunny day) could be due to the different sensor types (ASD FielSpec3 and Gamaya), the types of distributed variables and their ranges, and the number of correlated variables used in LUTreg.

### 5.2 Evaluation of the Retrieval Methods at Different Observation Dates

For the crop growing season, over the six observation dates, retrieval methods (LUTreg, hybrid, RF, and RFexp) were used to predict crop traits. There are three aspects that cannot be differentiated to study their effects on the estimates: Illumination variation, growth cycle, and the variation in structural crop traits (e.g., plant height, leaf orientation, and plant size).

For LAI, statistical methods (RF and RFexp) performed optimally under sunny conditions, as compared to LUTreg inversion and hybrid methods. However, under cloudy conditions and the late growing season dates (5 and 10 August), LUTreg inversion turned out to be the best. This indicates that statistical methods (RF and RFexp) were apparently more affected by illumination conditions than LUTreg and hybrid methods. The results obtained from statistical method (RFexp) under sunny conditions was consistent (NRMSE = 9.33% and RMSE = 0.32 m^2^/m^2^) with a previous study [91] that used UAV-hyperspectral vegetation index data (OSAVI) for a potato crop (RMSE of 0.67 m^2^/m^2^).

The best prediction for fCover was observed for the RFexp method under partial cloud conditions (27 July) at the maturity stage. Furthermore, the other methods (RF, LUTreg, and hybrid) also yielded the best predictions on that date (27 July). This observation was contradictory with our expectation, as the prediction of fCover under sunny conditions was not the best. This might partly be attributed to the impact of phenological growth stages on crop status, rather than the cloud shadow effect. In fact, using any retrieval method can yield an accurate result when the crop is near closure and covering the soil background. Our results (RMSE below 10%) are supported by a similar retrieval study [92], where the wide dynamic range vegetation index (WDRVI) delivered high accuracy with RMSE below 6% using hyperspectral data for corn. Likewise, for the CCC, the best estimate did not occur under sunny conditions (19 July), but on 14 July (using the statistical methods) or on 5 August (using LUTreg and hybrid methods), under variable illumination conditions. The unstable CCC result might have been introduced due to the uncertainty of the estimated LCC. Since Equation (1) was used in this study for converting the SPAD data to LCC values, a poor relationship between them was founded for potato crops (R^2^ = 0.46), as compared to other studied plant types [64].

We noticed that across the observation dates, LUTreg inversion could deliver generally superior results, compared to the hybrid method. These findings were expected, as a hybrid method inevitably is influenced by confounding factors, including: (1) LUT parameterization. Using *prior* knowledge from previous studies to fix some parameters (as mentioned previously) was possibly anticipated to lead to some error in the simulated canopy spectra. Thus, tuning the model input parameters (i.e., fB, D, hot, Zeta, and LIDF) are of tremendous importance in improving canopy simulations during crop development over time, as UAV-hyperspectral data has a very high spatial resolution (1.7 cm). At this resolution, the shadowing effect, cast by plants, and the bidirectional effects might have an impact. (2) ML optimization. Each algorithm has its own set of parameters; thus, they need to be tuned properly to having optimal values. Finally, (3) the proper choice of RTM type. The SLC radiative transfer model treats the canopy as a horizontally uniform layer (1D), which does not accommodate for row crops and, as such, does not fully represent the actual canopy architecture of the row-structure of the potato crop. For instance, at the early stage, the soil background is exposed within a plot, representing the vegetation in both the row and the furrow [79,93]. However, during the final stages, the potato crop completely covers the surface, leading to a homogeneous canopy. Hence, the model delivers a more accurate result. Nevertheless, the findings of LAI and fCover using the GPR and RF (hybrid methods; Figure 6) indicate that these methods achieved good accuracies, in terms of R^2^ and NRMSE (%), as opposed to CCF, when used for the CCC estimate. The latter might be affected due to the limitation of ML methods in predicting vegetation variables from remote sensing data [37,94] or the lack of accurate estimation of LCC obtained from the SPAD measurements.

The results revealed that the four retrieval methods—hybrid, LUTreg, RF, and RFexp—were affected by two main issues. First, the measured spectra still had some uncertainties. These may have originated from: (1) The exposure time, which was not adjusted carefully during the cloudy dates (i.e., 14 July); (2) instrumental issues, such as the spectral correction method was carried out for Gamaya to reduce the mismatch between ASD FieldSpec3 and UAV spectral band positions. This correction was not an accurate solution to solve the overlapping bandwidths of the spectral bands of Gamaya sensor. Second, uncertainties could have been induced by the in situ measurements. Using the LAI-2000 instrument often leads to LAI underestimation in the case of potato crops, as this instrument relies on several assumptions [95] which were violated in our case study. The assumptions of the LAI-2000 are that the potato leaves are small for the observed field of view (148° FOV), are an optical black in the wavelength region below 490 nm (i.e., not transmitting or reflecting incident radiation), and are randomly oriented with respect to azimuth [61]. For fCover, visual assessment is widely used in plant communities, due to its simplicity and rapid measurement; however, it can be a potential source of error, due to its subjective dependency and the difficulty in measuring the variable accurately [96]. Furthermore, when calculating the LCC from SPAD measurements using Equation (1), the transfer function should be calibrated to the particular crop of interest [97].

## 6 Conclusions

The purpose of this study was to evaluate the LUTreg-based inversion method integrated with the Cholesky method, in terms of providing improved LAI, fCover, and CCC estimates for a potato crop using UAV-hyperspectral data through the crop’s development over time. This study built upon an earlier study [30], where the selected method (LUTreg) was determined on a single observation date from field spectroradiometer measurements. We further compared hybrid and statistical methods, in order to investigate their performance through the growth season under variable illumination conditions. Information on the illumination condition was determined in the statistical method, to improve the accuracy of estimates.

Our major finding was that the LUTreg method was able to improve the accuracy of LAI and CCC, either using the whole data of the six observation dates or under different levels of nitrogen. However, the estimated fCover from LUTreg was improved slightly, as compared to LUTstd. Moreover, at each growth stage, LUTreg delivered superior accuracies, compared to the hybrid method. However, when comparing LUTreg with the statistical method (RF), the accuracy of LAI and fCover estimates decreased, while that for the CCC estimate did not. Finally, the use of exposure time as an explanatory variable in the RF method (RFexp) was successfully able to alleviate the influences of illumination variability during image acquisition and decreasing the errors in all predictions (i.e., LAI, fCover, and CCC).

These findings open an avenue for further studies. The use of the Cholesky method in LUTreg using PROSAIL [71], INFORM [98], and SCOPE [99] from remotely sensed data (e.g., Sentinel-2 and -3 imagery) needs further exploration for estimating other crop traits (e.g., nitrogen and biomass). In addition, future endeavors may include more calibration (e.g., radiometric correction) for UAV images acquired under cloudy conditions.

## Supplementary Material

Appendix

## Figures and Tables

**Table 1:** Flight conditions and camera settings for the campaigns in 2016.

Date	Growth Stage	Flight Time	SZA	SAA	Illumination	exp (Second)	
						VIS	NIR
8-July	Tuber bulking	12:00	28	165.58	Partial cloud cover	1/840	1/1135
14-July	Tuber bulking and flowering	12:30	29	165.51	Partial cloud cover	1/840	1/1135
19-July	Tuber bulking and flowering	12:15	30	165.62	Clear/ sunny	1/840	1/1135
27-July	Maturity	12:15	31	166.09	Partial cloud cover	1/496	1/840
5-August	Maturity	11:25	33	177.94	Full cloud cover	1/328	1/716
10-August	Maturity	11:46	35	162.3	Full cloud cover	1/328	1/552

**Note**: Solar zenith angle (SZA); solar azimuth angle (SAA); exposure time (exp); and visible and near-infrared bands (VIS and NIR, respectively).

**Table 2:** Input parameters of the Soil–Leaf–Canopy (SLC) model used for generating a look-up table (LUT).

Parameter	Unit	Range		Distribution	Fixed Value	Reference
		Min	Max			
**Leaf Parameter (PROSPECT-4)**						
Internal leaf structure, N	Unitless	1	2.5	Uniform		[78,79]
Chlorophyll content, LCC	(μg cm^–2^)	40	90	Gaussian *μ* = 65.36, *σ* = 9.38		From field measurement
Water content, Cw	(cm)				0.0317	[5]
Dry matter content, Cm	(gcm^−2^)				0.005	[79]
Senescence material, Cs	Unitless				0	From field experience
**Canopy Parameter (4SAIL2)**						
Leaf area index, LAI	(m^2^ m^–2^)	0.05	7	Gaussian *μ* = 2.85, *σ* = 1.17		From field measurement
Leaf inclination distribution functions (LIDFa and LIDFb)	Unitless				LIDFa (0.66), LIDFb (-0.04)	[30]
Hotspot coefficient, hot	(m m^–1^)				0.05	[80]
Vertical crown cover, Cv	Unitless	0.05	1	Gaussian *μ*= 0.71, *σ*= 0.23		[30]
Tree shape factor, zeta	Unitless				1	From field experience
Layer dissociation factor, D	Unitless				1	From field experience
Fraction of brown canopy area, fB	Unitless				0	From field experience
**Soil parameters (Hapke)**						
Hapke_b	Unitless				0.84	[66,72]
Hapke_c	Unitless				0.68	-
Hapke_h	Unitless				0.23	-
Hapke_B0	Unitless				0.3	-
Soil moisture, SM	Unitless				15	From field experience

**Note**: *μ* is the mean and *σ* is the standard deviation.

**Table 3:** List of selected ML methods implemented in ARTMO toolbox.

Algorithm	Brief Description	References
	**Non-Linear Non-Parametric Regression**	
Random Forest (Tree Bagger)	RF is an extension over bagging trees. In particular, random selection is applied to construct different subsets of training data sets, as well as their features, to grow trees instead of using all features. This leads to a consensus prediction.	[51]
Conical Correlation Forest	CCF is a member of the decision tree ensemble family. Conical correlation analysis is used to find feature projections, wherein a voting rule combines the predictions of individual conical correlation trees to make a final prediction for unknown samples.	[85,86]
Gaussian Process Regression	GPR, as one of the kernel-based regression methods, is a stochastic probability distribution-based process of estimation by providing the mean and covariance. Consequently, the confidence interval around the mean predictions can be provided to assess the uncertainties.	[87]

**Table 4:** Descriptive statistics of the measured variables over six dates in 2016 for a potato crop.

Var	Stats.	8 July	14 July	19 July	27 July	05 August	10 August	All Data
		Tuber Bulking	Tuber Bulking and Flowering	Tuber Bulking and Flowering	Maturity	Maturity	Maturity	
LAI (m^2^/m^2^)	Mean	1.91	2.19	2.22	2.98	3.94	3.69	2.85
	Min	0.19	0.06	0.56	0.92	1.64	2.35	0.06
	Max	2.84	3.74	4.04	5.25	6.67	5.46	6.67
	Stdev	0.62	0.91	0.86	0.99	1.05	0.77	1.17
	C.V.	0.32	0.42	0.39	0.33	0.27	0.21	0.41
fCover	Mean	0.47	0.58	0.62	0.77	0.91	0.88	0.71
	Min	0.05	0.15	0.1	0.35	0.55	0.7	0.05
	Max	0.65	0.85	0.95	0.95	1	1	1
	Stdev	0.17	0.22	0.25	0.14	0.12	0.09	0.23
	C.V.	0.36	0.38	0.41	0.19	0.13	0.11	0.33
CCC (g/m^2^)	Mean	1.37	1.48	1.66	1.93	2.27	2.22	1.84
	Min	0.15	0.05	0.38	0.48	0.81	1.18	0.05
	Max	2.1	2.89	3.3	3.62	3.85	3.63	3.85
	Stdev	0.47	0.68	0.77	0.78	0.69	0.6	0.75
	C.V.	0.35	0.46	0.46	0.4	0.31	0.27	0.41

**Note**: Var. are the variables of interest; Stdev is the standard deviation; Min is the minimum value; Max is the maximum value; C.V. is the coefficient of variation; LAI is the leaf area index; CCC is the canopy chlorophyll content; and fCover is the fractional vegetation cover.

**Table 5:** Accuracy assessment between hybrid method-based ML approaches for the whole growth season.

Methods	Stats.	LAI(m^2^/m^2^)	fCover	CCC(g/m^2^)
RF	R^2^	0.77	**0.82**	0.81
	NRMSE(%)	10.59	**10.59**	15.06
CCF	R^2^	0.59	0.65	**0.55**
	NRMSE(%)	11.59	16.58	**13.40**
GPR	R^2^	**0.70**	0.68	0.60
	NRMSE(%)	**9.80**	17.58	17.26

**Note**: The results of ground validation obtained from a hybrid method based on the best sample size, where the bold number indicates the best results; Stats is the statistical measures; R^2^ is the coefficient of determination; and NRMSE (%) is a normalized root mean square error.

**Table 6:** The coefficient of determination (R^2^) and Normalized Root Mean Square Error (NRMSE %) values obtained from different retrieval strategies.

			Different Retrieval Strategies							
Estimations	Growth Seasons	Illumination	Hybrid		LUTreg		RF		RFexp	
			R^2^	NRMSE	R^2^	NRMSE	R^2^	NRMSE	R^2^	NRMSE
LAI(m^2^/m^2^)	8 July (Tuber bulking)	Partial cloud cover	0.56	23.23	0.73	13.81	0.70	12.64	0.8	12.27
	14 July (Tuber bulking and flowering)	Partial cloud cover	0.64	15.26	0.71	14.83	0.65	16.23	0.76	14.69
	19 July (Tuber bulking and flowering)	Clear/Sunny	0.83	16.66	0.73	13.87	**0.87**	**9.33**	**0.88**	**8.11**
	27 July (Maturity)	Partial cloud cover	0.52	17.35	0.59	14.57	0.58	14.54	0.71	11.59
	5 August (Maturity)	Full cloud cover	**0.61**	**15.15**	**0.70**	**12.09**	0.46	15.50	0.63	11.81
	10 August (Maturity)	Full cloud cover	0.16	33.95	0.26	24.53	0.25	25.27	0.43	14.25
	All data	-	0.70	9.80	0.77	9.18	0.80	5.51	**0.83**	**5.36**
fCover	8 July (Tuber bulking)	Partial cloud cover	0.41	22.92	0.75	14.37	0.7	15.61	0.76	13.82
	14 July(Tuber bulking and flowering)	Partial cloud cover	0.64	19.92	0.77	17.12	0.72	17.5	0.79	13.71
	19 July (Tuber bulking and flowering)	Clear/Sunny	0.71	14.35	0.74	14.99	0.77	14.46	0.80	13.14
	27 July (Maturity)	Partial cloud cover	**0.55**	**13.97**	**0.74**	**12.80**	**0.740**	**12.53**	**0.86**	**8.03**
	5 August (Maturity)	Full cloud cover	0.38	13.96	0.71	13.76	0.66	21.89	0.91	8.81
	10 August (Maturity)	Full cloud cover	0.11	33.42	0.12	33.06	0.45	36.56	0.71	10.93
	All data	-	0.82	10.59	0.83	10.46	0.85	6.23	**0.86**	**5.87**
CCC(g/m^2^)	8 July (Tuber bulking)	Partial cloud cover	0.64	26.12	0.6	18.05	0.61	17.20	0.68	15.49
	14 July(Tuber bulking and flowering)	Partial cloud cover	0.68	16.83	0.6	17.09	**0.65**	**15.59**	**0.75**	**13.35**
	19 July (Tuber bulking and flowering)	Clear/Sunny	0.79	15.83	0.64	16.11	0.80	17.32	0.85	13.66
	27 July (Maturity)	Partial cloud cover	0.52	18.25	0.62	16.92	0.32	18.15	0.52	15.04
	5 August (Maturity)	Full cloud cover	**0.55**	**15.59**	**0.54**	**14.49**	0.47	19.28	0.56	14.53
	10 August (Maturity)	Full cloud cover	0.08	30.86	0.09	23.33	0.11	22.34	0.44	15.12
	All data	-	0.55	13.40	**0.62**	**12.16**	0.65	16.21	0.61	15.01

**Note**: The highlighted numbers indicate the best estimates for retrieval methods: For LAI, using the Hybrid method using Gaussian Process Regression (GPR); for fCover, using Random Forest (RF); and, for CCC, using Conical Correlation Forest (CCF) and LUTreg-based inversion (LUTreg).

## Abbreviations

The following abbreviations are used in this manuscript:

ASD FieldSpec3: Analytical Spectral Devices
ARTMO: Automated Radiative Transfer Models Operator
C.V.: Coefficient of Variation
DART: Discrete Anisotropic Radiative Transfer
DN: Digital number
ENVI: Environment for Visualizing Images
exp: exposure time
ELC: empirical line calibration
FWHM: Full Width at Half Maximum
FOV: field of view
FV2000: File Viewer software
GPS: Global Position System
INFORM: INvertible FOrest Reflectance Model
LIDFa: the average leaf slope
LIDFb: the distribution’s bimodality
LIDF: Leaf Inclination Distribution Function
NIR: Near-Infrared Range of spectrum
OSAVI: Optimized Soil-Adjusted Vegetation Index
PROSAIL: PROSPECT (leaf optical PROperties SPECTra model) and SAIL (Scattering by Arbitrarily Inclined Leaves)
PCA: Plant Canopy Analyzer
RTK-GPS: Real-Time Kinematic Global Positioning System
RGB: Red-Green-Blue
SLC: Soil–Leaf–Canopy
SCOPE: Soil Canopy Observation, Photochemistry, and Energy fluxes
SPAD: Soil Plant Analysis Development
SZA: solar zenith angle
SAA: solar azimuth angle
UAV: unmanned aerial vehicle
UTM31N: Universal Transverse Mercator Grid Zones 31North
VIS: Visible range of spectrum
VNIR: Visible and Near-Infrared Ranges
WDRVI: Wide Dynamic Range Vegetation Index
WGS84: World Geodetic System 1984
