# Supplementary material for: Comparison of Crop Trait Retrieval Strategies Using UAV-Based VNIR Hyperspectral Imaging
Source: Remote Sens (Basel). Author manuscript; Available in PMC 2022 Sep 7. (PMC7613394; doi:10.3390/rs13091748)
Supplement: Appendix [file EMS152667-supplement-Appendix.pdf]

Appendix A

Table A1. Radiometric accuracies across bands per date of the ELC for hyperspectral UAV orthomosaics.

Observation Dates		8 July Day1		14 July Day2		19 July Day3		27 July Day4		5 August Day5		10 August Day6	
Band No.	Band (nm)	R^2	RMSE	R^2	RMSE	R^2	RMSE	R^2	RMSE	R^2	RMSE		RMSE
1	474	0.99	0.0094	0.792	0.0422	0.9977	0.0044	0.9581	0.0183	0.982	0.0126	0.9634	0.018
2	483	0.9871	0.0106	0.8407	0.0367	0.999	0.003	0.9579	0.0181	0.9816	0.0127	0.9629	0.018
3	492	0.9842	0.0117	0.9236	0.0254	0.9997	0.0022	0.9633	0.0166	0.9814	0.0127	0.9619	0.018
4	501	0.9829	0.0121	0.9775	0.0142	0.9998	0.0029	0.9792	0.0121	0.9821	0.0125	0.9621	0.018
5	509	0.9838	0.0118	0.9914	0.009	0.9989	0.0051	0.9942	0.0067	0.9832	0.0122	0.9633	0.0179
6	518	0.9855	0.0112	0.9926	0.0081	0.9961	0.008	0.9883	0.0109	0.9842	0.0118	0.9649	0.0175
7	527	0.9868	0.0107	0.9925	0.0079	0.9905	0.0111	0.9588	0.0192	0.9848	0.0116	0.9661	0.0172
8	536	0.9871	0.0105	0.9921	0.008	0.9854	0.0134	0.9257	0.0252	0.9849	0.0114	0.9669	0.017
9	545	0.9869	0.0105	0.9913	0.0083	0.9835	0.014	0.9101	0.0273	0.9851	0.0113	0.9674	0.0168
10	554	0.9868	0.0106	0.99	0.0089	0.9853	0.013	0.9167	0.0257	0.9855	0.0111	0.9679	0.0166
11	569	0.9863	0.0106	0.9874	0.01	0.9897	0.0109	0.9322	0.0227	0.9856	0.0109	0.9682	0.0163
12	582	0.9857	0.0106	0.9838	0.0112	0.9935	0.009	0.9415	0.021	0.9855	0.0108	0.9681	0.0161
13	596	0.9854	0.0106	0.9785	0.0128	0.9963	0.0071	0.944	0.0205	0.9854	0.0107	0.9683	0.0158
14	610	0.9852	0.0106	0.9697	0.0152	0.9985	0.0048	0.9447	0.0203	0.9856	0.0106	0.969	0.0155
15	624	0.9845	0.0108	0.957	0.018	0.9995	0.0026	0.9444	0.0201	0.9855	0.0105	0.9697	0.0152
16	638	0.9836	0.011	0.9474	0.0197	0.9986	0.0026	0.9386	0.0208	0.9853	0.0105	0.9703	0.015
17	651	0.9811	0.0117	0.9461	0.0198	0.9979	0.0032	0.9447	0.0195	0.9855	0.0103	0.9711	0.0148
18	665	0.9762	0.0131	0.9518	0.0186	0.9986	0.003	0.967	0.0147	0.9867	0.0098	0.9725	0.0142
19	674	0.9706	0.0144	0.9619	0.0164	0.9996	0.0023	0.9838	0.01	0.9878	0.0093	0.9739	0.0137
20	682	0.968	0.0149	0.9723	0.0139	0.9996	0.0021	0.9889	0.0083	0.9875	0.0092	0.9741	0.0135
21	691	0.9829	0.038	0.9978	0.0136	0.9988	0.0093	0.998	0.0128	0.9976	0.0146	0.996	0.0187
22	699	0.9868	0.0335	0.9984	0.0117	0.9986	0.0096	0.9982	0.0115	0.9983	0.0119	0.9972	0.0157
23	708	0.9902	0.0287	0.9987	0.0105	0.9992	0.0077	0.9965	0.0171	0.9984	0.0117	0.9969	0.0163
24	716	0.9922	0.0248	0.9988	0.01	0.9996	0.0065	0.9877	0.0322	0.9984	0.0113	0.9965	0.0168
25	725	0.9882	0.0294	0.9988	0.0092	0.9998	0.0053	0.9338	0.0722	0.9982	0.0113	0.9956	0.0173
26	743	0.9845	0.0305	0.9981	0.0097	0.9995	0.0055	0.8446	0.1088	0.998	0.0111	0.9949	0.0156
27	761	0.9884	0.0237	0.998	0.0109	0.9995	0.0056	0.878	0.0937	0.9977	0.0111	0.9952	0.0135
28	779	0.987	0.0264	0.9988	0.0102	0.9996	0.0076	0.8976	0.087	0.9975	0.012	0.996	0.0131
29	797	0.9684	0.048	0.9988	0.0092	0.9997	0.0037	0.7959	0.1276	0.9981	0.0111	0.9961	0.0146
30	815	0.9747	0.0441	0.9984	0.0103	0.9999	0.0028	0.8049	0.126	0.9984	0.0102	0.9965	0.0144
31	825	0.9902	0.0275	0.9985	0.0102	0.9995	0.0073	0.9358	0.0726	0.9986	0.0094	0.9972	0.0129
32	835	0.9892	0.0293	0.9988	0.0092	0.9993	0.0081	0.9426	0.0683	0.9985	0.01	0.9972	0.0133
33	845	0.979	0.0406	0.9984	0.0105	0.9997	0.0061	0.862	0.1055	0.9978	0.0121	0.9965	0.0154
34	855	0.9851	0.0341	0.9984	0.0106	0.9996	0.0065	0.98	0.0417	0.9978	0.0124	0.9966	0.0156
35	865	0.992	0.0251	0.9987	0.0091	0.9997	0.0065	0.9906	0.0253	0.9981	0.0116	0.9973	0.014
36	875	0.9882	0.0309	0.9987	0.0089	0.9994	0.0082	0.986	0.0333	0.9986	0.0099	0.9979	0.0122
37	885	0.9882	0.0306	0.9978	0.0124	0.9985	0.0118	0.9921	0.0247	0.9985	0.0102	0.9976	0.0138
38	895	0.9887	0.0297	0.9975	0.0135	0.9997	0.0039	0.9687	0.0505	0.9973	0.0138	0.9971	0.0145
39	905	0.993	0.0229	0.9979	0.012	0.9989	0.0106	0.9428	0.0676	0.9982	0.0116	0.9966	0.0151
40	915	0.9943	0.0204	0.9987	0.01	0.9995	0.0058	0.968	0.0495	0.9986	0.0103	0.9972	0.0122
**Total mean**		0.985	0.0215	0.9777	0.014	0.9974	0.0066	0.9446	0.041	0.9914	0.0113	0.9821	0.0155

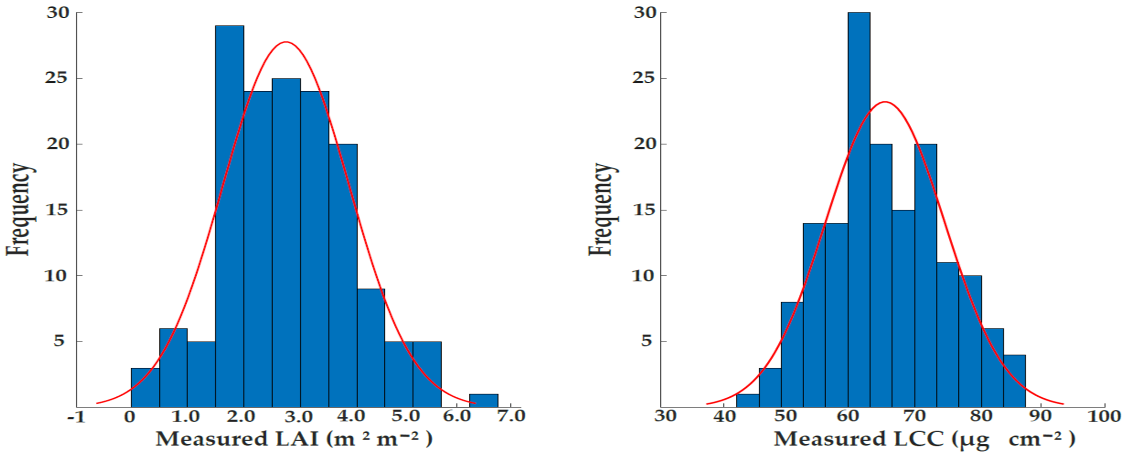

Figure A1. The distribution of measured leaf area index (LAI) and leaf chlorophyll content (LCC) using all data (156 samples).

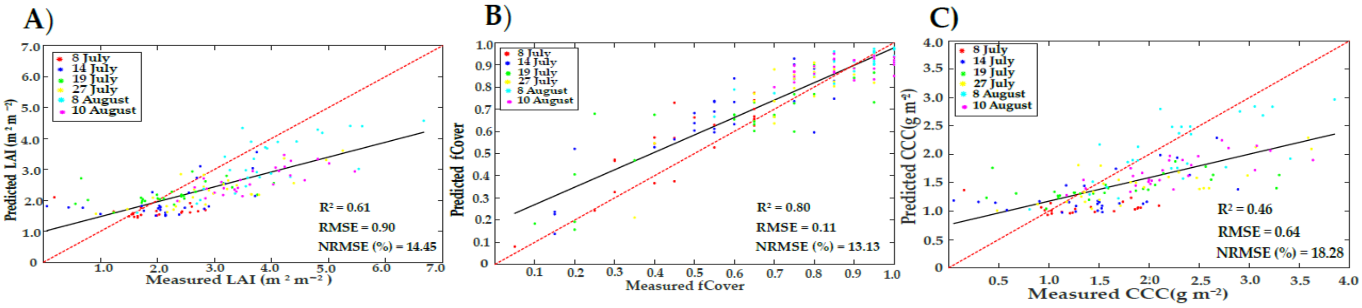

Figure A2. Scatterplots of LAI (**A**), fCover (**B**), and CCC (**C**) obtained from LUTstd using all data (156 samples); and the trend lines for linear fitting (black) and 1:1 line (dashed red).

Table A2. Average NRMSE % of 10 replicates based on cross-validation and ground validation under different sample sizes and machine learning methods for LAI estimates.

MLRAs	RF		CCF		GPR	
Samples	CV	GV	CV	GV	CV	GV
100	7.07 ^{g}	15.96 ^{i}	7.38 ^{f}	12.58 ^{e}	6.40 ^{e}	**9.80 ^{a}**
200	7.56 ^{h}	14.20 ^{g}	7.58 ^{g}	12.21 ^{b}	7.23 ^{f}	10.99 ^{d}
250	7.02 ^{g}	14.54 ^{h}	6.88 ^{e}	13.97 ^{g}	6.53 ^{f}	12.99 ^{d}
500	6.20 ^{f}	13.13 ^{f}	6.55 ^{d}	12.56 ^{d}	6.20 ^{c}	10.33 ^{b}
1000	5.18 ^{e}	12.01 ^{e}	6.52 ^{d}	12.94 ^{g}	6.20 ^{c}	15.39 ^{h}
2000	4.63 ^{d}	11.46 ^{d}	6.36 ^{c}	12.30 ^{c}	**6.09 ^{b}**	13.28 ^{f}
2500	4.48 ^{c}	**10.59 ^{a}**	6.53 ^{d}	**11.59 ^{a}**	6.29 ^{d}	12.10 ^{d}
3000	4.38 ^{b}	11.42 ^{d}	6.50 ^{d}	12.85 ^{f}	6.21 ^{c}	14.06 ^{g}
4000	4.06 ^{a}	10.90 ^{c}	**6.15 ^{a}**	12.83 ^{f}	**5.86 ^{a}**	13.00 ^{e}
5000	3.98 ^{a}	10.70 ^{b}	6.24 ^{b}	12.94 ^{g}	5.96 ^{b}	10.83 ^{c}

Note: The highlighted numbers indicate the best retrieval; Cv and Gv denote the cross-validation and ground validations; the letters represent the ranking of Friedman's aligned post-hoc test, there is no significant difference when sharing the same letter.

Table A3. Average NRMSE % of 10 replicates, based on cross-validation and ground validation under different sample sizes and machine learning methods, for fCover estimates.

MLRAs	RF		CCF		GPR	
**Samples**	**CV**	**GV**	**CV**	**GV**	**CV**	**GV**
100	2.70 ^{g}	12.58 ^{f}	1.49 ^{d}	17.03 ^{e}	3.72 ^{e}	17.58 ^{b}
200	2.23 ^{f}	11.51 ^{e}	2.58 ^{f}	16.65 ^{b}	1.45 ^{d}	**17.49 ^{a}**
250	2.26 ^{f}	11.16 ^{c}	2.24 ^{f}	16.95 ^{d}	1.37 ^{c}	18.20 ^{d}
500	1.97 ^{e}	**10.59 ^{a}**	1.90 ^{e}	**16.58 ^{a}**	1.41 ^{d}	17.96 ^{c}
1000	1.67 ^{d}	10.83 ^{a}	1.61	16.84 ^{c}	1.32 ^{b}	18.48 ^{e}
2000	1.54 ^{c}	11.22 ^{d}	1.46 ^{d}	17.33 ^{g}	1.30 ^{b}	21.29 ^{i}
2500	1.51 ^{c}	11.06 ^{b}	1.42 ^{c}	17.13 ^{f}	1.30 ^{b}	20.73 ^{h}
3000	1.46 ^{b}	12.07	1.40 ^{c}	17.08 ^{d}	1.29 ^{b}	18.90 ^{f}
4000	**1.41 ^{a}**	11.01 ^{b}	**1.37 ^{a}**	17.29 ^{h}	**1.25 ^{a}**	20.37 ^{g}
5000	1.42 ^{b}	11.29 ^{d}	1.38 ^{b}	17.14 ^{e}	1.29 ^{b}	18.24 ^{d}

Note: The highlighted numbers indicate the best retrieval; Cv and Gv denote the cross-validation and ground validations; the letters represent the ranking of Friedman's aligned post-hoc test, there is no significant difference when sharing the same letter.

Table A4. Average NRMSE % of 10 replicates, based on cross-validation and ground validation under different sample sizes and machine learning methods, for CCC estimates.

MLRAs	RF		CCF		GPR	
**Samples**	**CV**	**GV**	**CV**	**GV**	**CV**	**GV**
100	7.85 ^{i}	30.45 ^{i}	8.00 ^{g}	14.20 ^{f}	7.63 ^{h}	18.21 ^{b}
200	8.93 ^{j}	26.85 ^{h}	8.58 ^{h}	15.94 ^{g}	8.17 ^{i}	29.90 ^{i}
250	7.77 ^{h}	27.84 ^{g}	7.69 ^{f}	13.85 ^{e}	7.25 ^{g}	30.47 ^{j}
500	6.57 ^{g}	23.17 ^{f}	6.93 ^{c}	14.91 ^{g}	6.60 ^{c}	**17.26 ^{a}**
1000	5.78 ^{f}	22.61 ^{e}	7.13	**13.40 ^{a}**	6.86 ^{f}	20.99 ^{h}
2000	5.01 ^{e}	20.87 ^{d}	6.99 ^{d}	13.49 ^{b}	6.69 ^{d}	19.92 ^{e}
2500	4.81 ^{d}	**15.06 ^{a}**	7.09 ^{e}	17.13 ^{i}	6.83 ^{f}	20.73 ^{f}
3000	4.66 ^{c}	19.07 ^{b}	7.06 ^{e}	**13.44 ^{a}**	6.75 ^{e}	19.86 ^{d}
4000	4.40 ^{b}	20.05 ^{d}	**6.77 ^{a}**	13.66 ^{d}	**6.45 ^{a}**	20.91 ^{g}
5000	**4.32 ^{a}**	19.92 ^{c}	6.89 ^{b}	13.53 ^{c}	6.59 ^{b}	19.84 ^{c}

Note: The highlighted numbers indicate the best retrieval; Cv and Gv denote the cross-validation and ground validations; the letters represent the ranking of Friedman's aligned post-hoc test, there is no significant difference when sharing the same letter.

References

1. Tao, H.; Feng, H.; Xu, L.; Miao, M.; Long, H.; Yue, J.; Li, Z.; Yang, G.; Yang, X.; Fan, L. Estimation of crop growth parameters using UAV-based hyperspectral remote sensing data. *Sensors* **2020**, *20*, 1296. [CrossRef]
2. Cilia, C.; Panigada, C.; Rossini, M.; Meroni, M.; Busetto, L.; Amaducci, S.; Boschetti, M.; Picchi, V.; Colombo, R. Nitrogen status assessment for variable rate fertilization in maize through hyperspectral imagery. *Remote Sens.* **2014**, *6*, 6549–6565. [CrossRef]
3. Verger, A.; Martínez, B.; Camacho-de Coca, F.; García-Haro, F. Accuracy assessment of fraction of vegetation cover and leaf area index estimates from pragmatic methods in a cropland area. *Int. J. Remote Sens.* **2009**, *30*, 2685–2704. [CrossRef]
4. Gitelson, A.A.; Keydan, G.P.; Merzlyak, M.N. Three-band model for noninvasive estimation of chlorophyll, carotenoids, and anthocyanin contents in higher plant leaves. *Geophys. Res. Lett.* **2006**, *33*. [CrossRef]
5. Clevers, J.G.; Kooistra, L. Using hyperspectral remote sensing data for retrieving canopy chlorophyll and nitrogen content. *IEEE J. Sel. Top. Appl. Earth Obs. Remote Sens.* **2011**, *5*, 574–583. [CrossRef]
6. Hoeppner, J.M.; Skidmore, A.K.; Darvishzadeh, R.; Heurich, M.; Chang, H.C.; Gara, T.W. Mapping canopy chlorophyll content in a temperate forest using airborne hyperspectral data. *Remote Sens.* **2020**, *12*, 3573. [CrossRef]
7. Cheng, T.; Lu, N.; Wang, W.; Zhang, Q.; Li, D.; YAO, X.; Tian, Y.; Zhu, Y.; Cao, W.; Baret, F. Estimation of nitrogen nutrition status in winter wheat from unmanned aerial vehicle based multi-angular multispectral imagery. *Front. Plant Sci.* **2019**, *10*, 1601.
8. Shang, J.; McNairn, H.; Schulthess, U.; Fernandes, R.; Storie, J. Estimation of crop ground cover and leaf area index (LAI) of wheat using RapidEye satellite data: Prelimary study. In *Proceedings of the 2012 First International Conference on Agro-Geoinformatics (Agro-Geoinformatics)*; IEEE: New York, NY, USA, 2012; pp. 1–5.
